# Cancer Gene Prioritization for Targeted Resequencing Using FitSNP Scores

**DOI:** 10.1371/journal.pone.0031333

**Published:** 2012-03-01

**Authors:** Annelies Fieuw, Bram De Wilde, Frank Speleman, Jo Vandesompele, Katleen De Preter

**Affiliations:** Center for Medical Genetics, Ghent University Hospital, Ghent, Belgium; Tor Vergata University of Rome, Italy

## Abstract

**Background:**

Although the throughput of next generation sequencing is increasing and at the same time the cost is substantially reduced, for the majority of laboratories whole genome sequencing of large cohorts of cancer samples is still not feasible. In addition, the low number of genomes that are being sequenced is often problematic for the downstream interpretation of the significance of the variants. Targeted resequencing can partially circumvent this problem; by focusing on a limited number of candidate cancer genes to sequence, more samples can be included in the screening, hence resulting in substantial improvement of the statistical power. In this study, a successful strategy for prioritizing candidate genes for targeted resequencing of cancer genomes is presented.

**Results:**

Four prioritization strategies were evaluated on six different cancer types: genes were ranked using these strategies, and the positive predictive value (PPV) or mutation rate within the top-ranked genes was compared to the baseline mutation rate in each tumor type. Successful strategies generate gene lists in which the top is enriched for known mutated genes, as evidenced by an increase in PPV. A clear example of such an improvement is seen in colon cancer, where the PPV is increased by 2.3 fold compared to the baseline level when 100 top fitSNP genes are sequenced.

**Conclusions:**

A gene prioritization strategy based on the fitSNP scores appears to be most successful in identifying mutated cancer genes across different tumor entities, with variance of gene expression levels as a good second best.

## Introduction

Currently, cancer exome and genome sequencing is technically possible through next generation sequencing technologies that provide high throughput and low cost per base compared to classical Sanger sequencing [1]. However, due to the massive amount of sequence data generated on both coding and non-coding genomic regions, a challenge for the identification of disease relevant mutations or variations arises. Moreover, due to the high overall cost of these new technologies, such a genome wide screen is typically performed on a limited number of samples, which reduces the statistical power of such studies. Therefore, targeted resequencing is still being performed and remains a relevant and valid method that can circumvent these issues [2]. By focusing on specific candidate genes, a larger cohort of samples can be screened, which will increase the statistical power of the data analysis and will allow a better discrimination between driver and passenger mutations. The subsequent reduction in the amount of generated sequence information, often accompanied with higher coverage depth, will significantly facilitate the handling and interpretation of the data.

Crucially, such a targeted approach requires a method to prioritize and rationally select suitable candidate genes to include in the sequencing effort. This study aimed at the evaluation of four different strategies to prioritize candidate genes for targeted resequencing of cancer genomes.

A first approach is based on the fitSNP (functionally interpolating single nucleotide polymorphism) database, containing differential expression ratio (DER) values for over 18,000 human protein coding genes [3]. These DER values are calculated based on mRNA gene expression studies in the GEO (gene expression omnibus) database [4] and represent the ratio between the number of studies in which a gene is found to be differentially expressed and the number of studies in which the gene expression has been evaluated. Genes with DER values higher than 0.55 appear to be associated with the occurrence of disease associated variants [3]. Here, we hypothesize that the DER value of a gene can be used to predict the presence of mutations in cancer genomes.

The second prioritization strategy is related to the fitSNP approach and is based on the actual variance of the gene expression levels within one tumor entity (calculated as the standard deviation in one particular data set). This hypothesis is based on the idea that the variance in gene expression is caused by one or more perturbation mechanisms, including gene mutations.

The correlation coefficient between gene expression levels and gene copy numbers was evaluated as a third strategy, allowing the identification of dosage sensitive genes. Our hypothesis states that dosage sensitive genes are more prone to acquire mutations that can deregulate their expression and function.

The final strategy is linked to the Knudson-two hit hypothesis that states that tumor suppressor genes are biallelically inactivated [5]. We therefore explored whether genes with a high frequency of copy number loss (first hit) across the data set are more likely to carry a mutation (second hit).

In this study, we specifically evaluated whether the top-ranked genes in the prioritized gene lists are more likely to carry somatically acquired mutations. Apart from candidate gene ranking based on a single prioritization strategy, we also explored if combinations could improve the original results. Publically available data sets were used, consisting of copy number, gene expression and mutational data for six different tumor types: breast cancer, colon cancer, pancreas cancer, ovarian cancer, glioblastoma and medulloblastoma. Table 1 provides an overview of the different studies and number of samples available for the different information layers.

**Table 1:** Overview of the publically available tumor data sets, used in this study.

Tumor entity	Copy number data	Gene expression data	Combined CN and GE data	Gene mutational data
**Breast cancer**	22 samples	15 samples	12 samples	10 primary breast ductal adenocarcinomas
	GSE22840 (GSE22839)	GSE22840 (GSE22544)		±18000 genes sequenced
				Sjöblom *et al.* [10], Wood *et al.* [11]
**Colon cancer**	38 samples	19 samples	19 samples	11 liver metastases from colorectal carcinomas
	GSE17047	GSE17047		±18000 genes sequenced
				Sjöblom *et al.* [10], Wood *et al.* [11]
**Ovarian cancer**	9 samples	9 samples	9 samples	7 ovarian clear cell carcinomas*
	GSE19539	GSE19539		±18000 genes sequenced
				Jones *et al.* [7]
**Glioblastoma**	15 samples	15 samples	15 samples	19 primary glioblastoma multiforme samples*
	GSE10878	GSE10878		±20000 genes sequenced
				Parsons *et al.* [6]
**Pancreas cancer**	30 samples	36 samples	/	24 primary/metastases pancreas adenocarcinomas
	GSE7599	GSE15471		±20000 genes sequenced
				Jones *et al.* [8]
**Medulloblastoma**	60 samples	77 samples	/	14 pediatric classic medulloblastomas
	GSE8634	GSE21140		±21000 genes sequenced
				Parsons *et al.* [9]

* *one sample was excluded from this study due to a hypermutated profile caused by chemotherapeutic treatment.*

*CN: copy number; GE: gene expression.*

## Results

### Comparison of the single prioritization strategies

**Figure 1:**
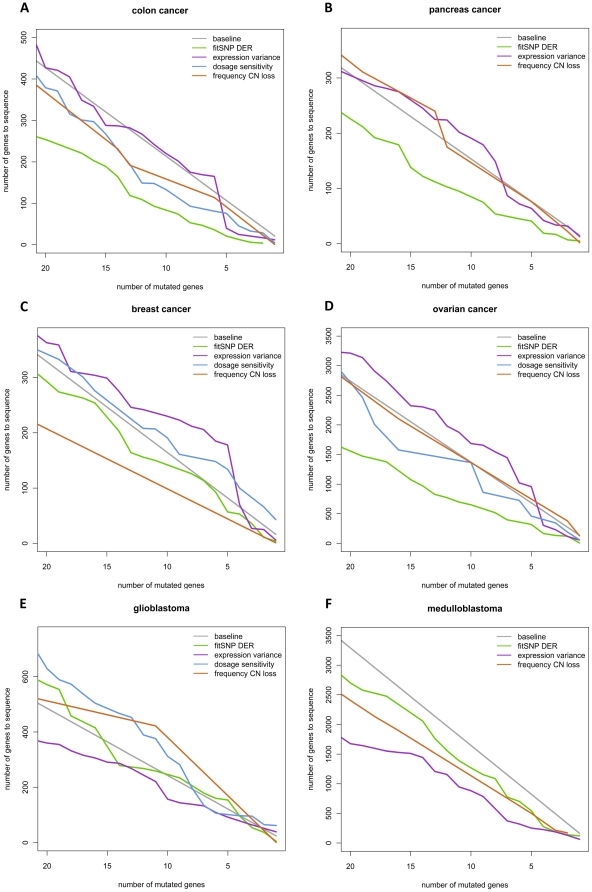
The number of mutated genes in relation to a certain number of top-ranked genes. Mutation plots showing the amount of genes that need to be sequenced (y-axis) in order to find a certain number of mutated genes (depicted on the x-axis), for the six different tumor types. A: colon cancer; B: pancreas cancer; C: breast cancer; D: ovarian cancer: E: glioblastoma; F: medulloblastoma.

The four single prioritization strategies are compared with each other and with the baseline PPV for the six different tumor types separately. The curves plotted in Figure 1 represent the number of genes that have to be sequenced to obtain a certain number of mutated genes. Curves below the baseline point at enrichment of mutation genes in the top ranked genes and indicate a valuable strategy for targeted resequencing.

The fitSNP curve for colon cancer clearly shows that prioritizing based on fitSNP DER values leads to a huge improvement in the discovery of mutated genes compared to a random selection of genes (Figure 1A). When 100 top fitSNP genes are sequenced, the PPV or mutation rate is increased by 2.3 fold compared to the baseline PPV (11% compared to 4.7%) (Table S1). This is also reflected in the number of genes that need to be sequenced to find 10 mutated genes; more than double the number of randomly selected genes (213) should be sequenced in comparison with 93 top fitSNP genes. Although fitSNP clearly outperforms the other prioritization strategies in colon cancer, the other three also successfully prioritize mutated genes, even though for the expression variance this improvement is only seen within the top 100 genes (Figure S1A). The maximum PPV that could be obtained for colon cancer is 50% for the top 4 fitSNP genes (Table S1).

Also for pancreas cancer, fitSNP strategy outperforms the other strategies and random selection (Figure 1B). For both the expression variance and the frequency of copy number loss no substantial improvement was noticed. Due to a lack of matching gene expression and copy number data for pancreas cancer, no dosage sensitivity values could be determined. The increase in PPV starts with larger gene lists in pancreas cancer compared to colon cancer and is already obvious for the top 250 genes. Eleven mutated genes can be found when the top-100 fitSNP genes are sequenced (PPV: 11%), compared to 6 genes with mutations for a random selection of 100 candidate genes (PPV: 6.5%), which is an almost 2-fold increase (Table S1). Within the top 7 of fitSNP ranked genes a maximum PPV of 28.6% was obtained (Table S1, Figure S1B).

For breast cancer the fitSNP strategy shows again an improvement compared to the baseline values, however this improvement is rather modest. For instance to find 10 mutated genes, 164 random genes should be sequenced compared to 150 top fitSNP genes (Figure 1C, Table S1). The expression variance strategy proves to be better than a random gene selection only when the top-50 genes are sequenced. The gene dosage sensitivity did not lead to any improvement in the results in this tumor type. For the top-100 fitSNP genes and top-50 expression variance genes a steep increase in PPV is present, with maximum PPV of 25% (top-4 genes) and 16% (top-6 genes) respectively (Table S1, Figure S1C).

Since the baseline PPV for ovarian cancer is very low (0.73%), more than 1300 random genes should have to be sequenced to find 10 genes with a mutation (Figure 1D). However, when focusing on the top fitSNP genes, only about half the number needs to be sequenced (651), confirming that the fitSNP strategy is also a valid strategy for this tumor type. To a lesser extent the gene dosage could also increase the number of mutated genes found for the same number of sequenced genes.

For the two remaining tumor types, glioblastoma and medulloblastoma, the expression variance rather than the fitSNP strategy seems to show the best results (Figure 1E, 1F). In glioblastoma the expression variance is the best strategy to improve mutation gene selection compared to the baseline, although when looking at the top-100 ranked genes, an increase in PPV can especially be seen for the fitSNP strategy (Figure S1E).

The baseline PPV in medulloblastoma could be improved using all three strategies. For instance, to find 10 mutated genes in a screen, more than 1600 random genes have to be sequenced, which can be decreased to 321, 416 and 445 top genes for expression variance, frequency of copy number loss and fitSNP DER values, respectively. Looking at the PPV plot for medulloblastoma a rapid decrease can be seen for the top-ranked genes of all strategies, indicating that none of the mutated genes can be found in either of the top-ranked gene lists (Figure S1F).

### Prioritizing using combined strategies

We occasionally observed substantial improvements in PPV when combining different strategies (Table S1, Figure S2). One clear example is breast cancer, where the mean PPV value reaches 26.9% for the top-25 genes when gene dosage sensitivity values (0% PPV for top-25 using single method) and the frequency of copy number loss (9.2% for top-25 using single method) are combined (Table S1, Figure S2C).

Another example is medulloblastoma where none of the three evaluated parameters had a PPV value higher than 0% for the top-50. Combinations of fitSNP DER value and expression variance or expression variance and frequency of copy number loss showed a clear increase in PPV value in the top-ranked genes (Table S1, Figure S2F).

For glioblastoma, fitSNP values in combination with expression variance clearly performed best; to find 10 mutated genes 120 top genes should be sequenced when the combined strategy is used, compared to 259 or 157 genes for the fitSNP or expression variance single strategies, respectively (Table S1, Figure S2E). These results indicate that improvements in PPV value can be obtained by using combinations of two different strategies. However, for some combinations, an impairment rather than improvement of the results was obtained, showing that combining different prioritizing strategies does not per se result in better candidate gene rankings.

### Comparison of all strategies across the different tumor entities

To compare the different prioritization methods across the six tumor entities, a weighted ranking method was applied on the mean of the PPV value of the 100 top-ranked genes, to produce a ranked list of prioritization methods (Table S2, Figure S3). The fitSNP DER value strategy in combination with the expression variance, was ranked as the best overall method for the prioritization of cancer genes for targeted resequencing, followed by the fitSNP DER values alone. Similar results were seen using a wide range of different cut-offs with respect to the number of top-ranking genes taken into account (Table S2).

The gene dosage sensitivity value was ranked last and was the only strategy that was ranked below the baseline value, indicating that this strategy on itself is not useful to prioritize mutated genes in the tested cancer entities. In contrast, when combined with either the fitSNP or the frequency of copy number loss strategy, the gene dosage sensitivity value was ranked third and fourth, respectively.

### Differences in mutational burden across the different tumor types

When looking at the mutation frequency curves for the six different tumor types (Figure 1A–F), we observe that for both ovarian cancer and medulloblastoma the baseline PPV or mutation rate is very low compared to the four other tumor entities (Table S1). The baseline PPV of pancreas cancer (6.5%) is more than 10 times higher than that of medulloblastoma (0.6%). This means that to find 10 genes with mutations about 150 random genes would have to be sequenced in pancreas cancer, but over 1500 in medulloblastoma (Figure 1C, 1F). The baseline PPV for breast cancer, colon cancer and glioblastoma are more comparable with pancreas cancer and lie between 4.1 and 6.1%, whereas ovarian cancer has a baseline PPV of 0.7%.

## Discussion

Given the current high costs of exome and whole genome sequencing, we assessed whether targeted resequencing of prioritized genes is a cost-efficient alternative to study a limited but relevant subset of putative cancer genes. Four main approaches to prioritize genes were evaluated: a gene's fitSNP DER value, the variance of gene expression levels within a tumor type, the gene dosage sensitivity and the frequency of gene copy number loss.

If a prioritization strategy is valid, the top-ranked genes in the ordered gene list should be enriched for mutated genes. We evaluated this enrichment by calculating the positive predictive value (PPV), which represents the sequencing yield as the fraction of mutated genes relative to the total number of genes analyzed. If mutated genes are enriched in the top-ranked genes, the PPV will increase for a smaller number of top-ranked genes. PPV values were calculated for decreasing numbers of top-ranked genes, and mean PPV values were calculated for a number of defined sizes of top-ranked gene lists. Besides an increase in PPV in relation to smaller gene lists, a valid strategy should also require a high PPV in comparison to the baseline, as this indicates that a high number of mutated genes can be expected when performing a targeted resequencing experiment.

Our results clearly demonstrate improvements in the mutation rate of the selected genes when prioritization strategies are used compared to the baseline level. These improvements are seen in several tumor types and using different prioritization strategies, with some variability between the different tumor types. This variability indicates that there is no universal method to prioritize genes across all tumor types, although the best improvements and the largest overall PPV values were obtained for the fitSNP strategy.

These between tumor entities differences are most likely reflecting the reported diversity in mutational landscape in the different tumor entities, as well as the specific mutational background of the individual tumor genomes [6], [7]. For instance, we noticed a very small mutation frequency for the pediatric cancer medulloblastoma, which is in concordance with the report of Parsons *et al.* describing that this tumor displays a very different mutational landscape compared to adult tumors [7]. Low mutation frequencies are not confined to pediatric cancers only, as in this specific study we found a mutation rate of only 0.7% in ovarian clear cell carcinoma samples, which was remarkably lower than in the other studied adult tumor types (4.1–6.5%).

**Figure 2:**
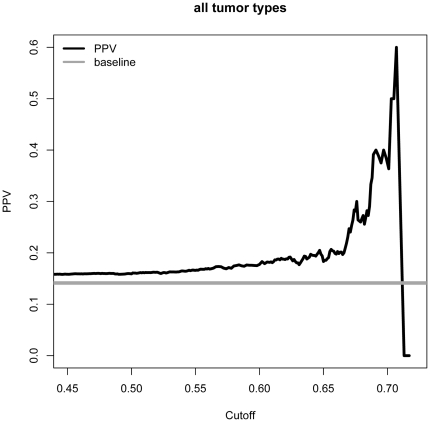
PPV plot of the fitSNP strategy for the combined tumor entities. A PPV plot for the fitSNP strategy, performed on the mutation data of all combined tumor entities, in function of different prioritization value cut-offs.

In order to evaluate the different methods across all tumor data sets, a weighted ranking method was used, pointing out that the best overall performing strategy was based on fitSNP differentially expressed ratio (DER) values. While it was previously hypothesized by Chen and colleagues that cancer genes have higher fitSNP DER values, the authors did not validate this by comparing fitSNP values to the mutational status of the genes [3]. From our results, we conclude that the cut-off for fitSNP DER values as determined in the original study (i.e. 0.55 [3]), is not optimal to prioritize mutated cancer genes, since for none of the tumors a substantial increase in PPV could be found when using this threshold. We suggest using a higher threshold of 0.65 for the prediction of variants in cancer genes (as can be deduced from Figure 2 which represents the overall PPV combining all tumor types).

The gene dosage sensitivity does not seem to have any prioritization value, whereas the expression variance and frequency of copy number loss were somewhat better than gene dosage sensitivity but less good than fitSNP. Of note, the frequency of copy number loss turned out to be not very useful in practice, since the low number of cut-off values prevents to make distinct gene selections, making the expression variance a preferred second best strategy.

By combining two different strategies, it was sometimes possible to improve the results of the individual strategies. Although the improvements could occasionally be huge, it again seemed to be highly dependent on the data set reflecting the different mutational mechanisms in different tumors. For instance, none of the three prioritization methods evaluated were useful for medulloblastoma, whereas combinations of two different parameters did successfully prioritize genes.

While various cancer gene prioritization methods were shown to be capable of increasing the yield of mutated cancer genes in the different tested cancer entities, none of the methods specifically enriched for genes that were mutated in more than one sample (data not shown). This is probably due to the limited number of cancer genomes studied within each entity and the fact that the majority of the genes are found to be mutated in only one sample (90 to 91%) (Table S3).

There are some limitations to this study that need to be considered, for instance the sample size of some of the data sets was rather limited, especially for the large genome sequencing studies (7–24 samples per entity). However, it is at this point difficult to find large cancer genome sequencing studies performed on an adequate number of samples, confirming the starting premise of our work that sequencing is currently cost prohibitive.

Due to the limited information that is presently available on driver and passenger genes, we could not properly investigate whether the fitSNP strategy is able to distinguish between driver and passenger mutations. However, the top-10 fitSNP genes contain 30% of Cancer Gene Census genes [8], i.e. RUNX1, TRA@ and NF1, whereas two other genes out of the top-10, CTNNA1 and SMAD3, have an established role in cancer development as well [9], [10], illustrating that this strategy helps to identify genes with proven role in carcinogenesis (Table S4, Figure S4).

In addition to the validity of the proposed strategy for targeted resequencing, gene prioritization could also be an added value to exome or whole genome sequencing. After such sequencing efforts on a limited cohort, the variants that are found will most probably have to be validated in a larger cohort. The fitSNP strategy might be helpful for prioritization and filtering of cancer genes in such a validation study.

## Materials and Methods

### Lists of mutated genes in cancer

Six large scale sequencing studies were used for the extraction of mutational data on six different tumor entities (breast cancer, colon cancer, pancreas cancer, ovarian cancer, glioblastoma, medulloblastoma) [7], [11]–[15] (Table 1). These data sets consist of sequencing information on approximately 18,000 to 21,000 genes, with a sample size ranging from 7 to 24, and were used to validate the different prioritization strategies. Hypermutated samples, due to chemotherapeutic treatment, as described in the respective papers [11], [13], were excluded from analysis.

### Copy number and gene expression data sets

For the six tumor entities copy number and gene expression data were downloaded from GEO [4]. We specifically selected samples with a tumor histology corresponding to that of the samples in the large scale sequencing screens as closely as possible (Table 1). For pancreas cancer and medulloblastoma no matching gene expression and copy number data were available. The sample sizes in these studies ranged from 9 to 77.

### Data analysis

For all copy number data sets, circular binary segmentation (CBS) values [16] were determined and extracted for each gene location. If no CBS value was available for a certain gene location, the nearest value was assigned to the gene. These CBS values were used to determine the frequency of copy number loss of each gene in the tumor cohort, and were correlated with the gene expression levels (Spearman rank correlation). For determination of copy number loss, different cut-off settings were used, according to the information provided in the original paper of the data set used (Table S5). The expression variance for each gene within each tumor type was calculated by the standard deviation of logged expression levels.

Based on their corresponding prioritization value, either fitSNP DER value [17], expression variance, gene dosage sensitivity or frequency of copy number loss, genes were ranked in descending order.

For the combined methods, the top-ranked gene lists were determined by taking the intersection of the top-ranked genes as defined by two single parameters.

For each cancer entity, the number of mutated genes was plotted (y-axis) in relation to a certain number of top-ranked genes that should be sequenced (x-axis). For the single prioritization strategies these curves were then compared with the baseline mutational level in the tumor entity, which represents the ratio of mutated genes versus the number of genes sequenced to obtain a certain number of mutated genes if no prioritization strategy is used.

In addition, the positive predictive value (PPV), or the mutation rate, was calculated for all different strategies. This value represents the ratio between the number of genes with mutations and the total number of genes in a certain gene subset. These values were calculated for different cut-off levels of top-ranked genes (500 - 400 - 300 - 200 - 150 - 100 - 75 - 50 - 25 - 10), whereby the change in PPV compared to the baseline value was evaluated.

In order to identify the best performing strategy across the different tumor types, mean PPV values determined for several top-ranked genes cut-offs, and the baseline PPV, (Table S1) were analysed using brute force weighted ranking analysis. This analysis yielded a ranked list of the different prioritization methods across the different tumor types (Table S2). For the weighted ranking analysis of the 10 different prioritization strategies (4 single strategies and 6 combined strategies), the medulloblastoma and pancreas cancer data sets were excluded, due to the absence of gene dosage sensitivity values.

As an evaluation of the fitSNP cut-off determined by Chen et al. [3], the mutational information on all different cancer types was combined and the PPV was determined for different fitSNP cut-off values (Figure 2).

For all analyses, the statistical platform R was used (packages GEOquery, DNAcopy, RankAggreg) [18]–[20].

## Supporting Information

Figure S1 **Overview of PPV plots in function of the number of sequenced genes for the six cancer entities.**
(TIF)

Figure S2 **Visualization of the weighted ranking results for the top-100 ranked genes.** The grey lines represent the ranking of the four different tumor types for the 10 prioritization strategies (4 single strategies and 6 combined strategies) and the baseline level. In red, the result of the brute force ranking algorithm is shown. The black line is where the baseline level is ranked across the different data sets. EV: expression variance; DS: dosage sensitivity; FCNL: frequency of copy number loss
(TIF)

Figure S3 **Plots of the number of mutated genes in relation to a certain number of top-ranked genes for the six cancer entities, including the combination strategies.**
(TIF)

Figure S4 **Plots of the percentage of mutated fitSNP genes that are found to be drivers.** For colon cancer, glioblastoma, pancreas cancer and breast cancer, the PPV is plotted for the top 500 fitSNP genes (black line). The grey line represents the percentage of mutated fitSNP genes that are identified as driver genes according to the respective publications. Enrichment of identified driver genes can be seen in the top fitSNP genes in both colon cancer and glioblastoma, whereas in pancreas cancer and breast cancer this could not be confirmed.
(PDF)

Table S1 **Overview of the analyses per cancer entity and prioritization strategy.** Overview listing the PPV, number of sequenced genes and number of mutated genes for the baseline PPV, maximum PPV, a different number of top-ranked genes and 1 to 20 mutated genes. In red, values are indicated that don't match with the number of top-ranked genes considered, due to cut-off restrictions of the prioritization method.
(XLS)

Table S2 **Ranked lists of the different prioritization methods.** These ranked lists are based on the brute force weighted ranking algorithm, performed across the different cancer entities. The ranking has been performed on the single prioritization strategies alone as well as together with the combined strategies.
(XLS)

Table S3 **Overview of the mutated genes in the different studied cancer entities.**
(XLS)

Table S4 **Overview of the mutated genes in the top-500 fitSNP genes.**
(XLSX)

Table S5 **Cut-offs used for the different cancer entities to determine copy number loss.**
(XLS)
