# Supplementary figures and images for: Cancer Gene Prioritization for Targeted Resequencing Using FitSNP Scores
Source: PLoS One. 2012 Mar 1;7(3):e31333. doi: 10.1371/journal.pone.0031333 (PMC3291573; doi:10.1371/journal.pone.0031333)

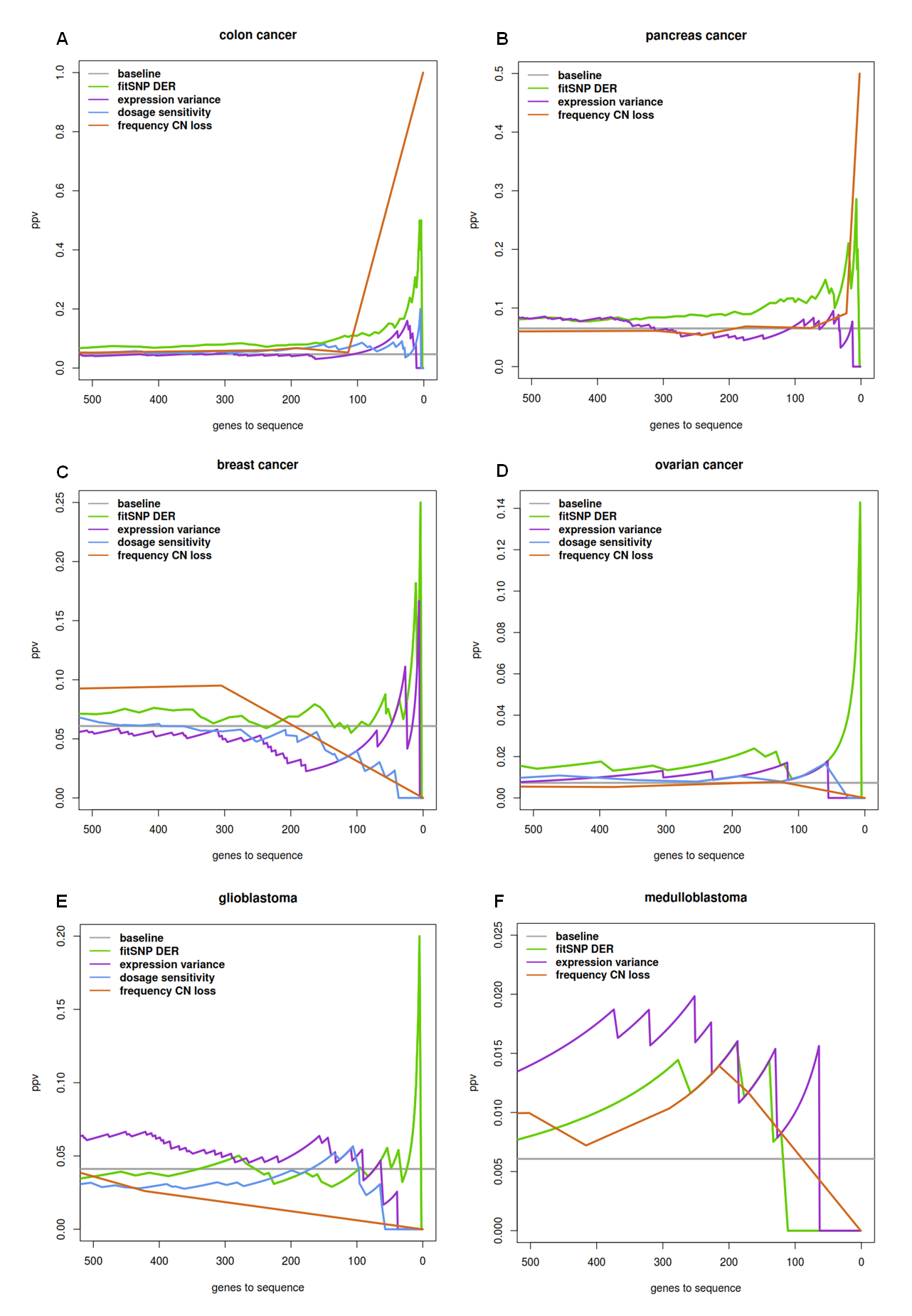

Supplement: Figure S1 — Overview of PPV plots in function of the number of sequenced genes for the six cancer entities. (TIF) [file pone.0031333.s001.tif]

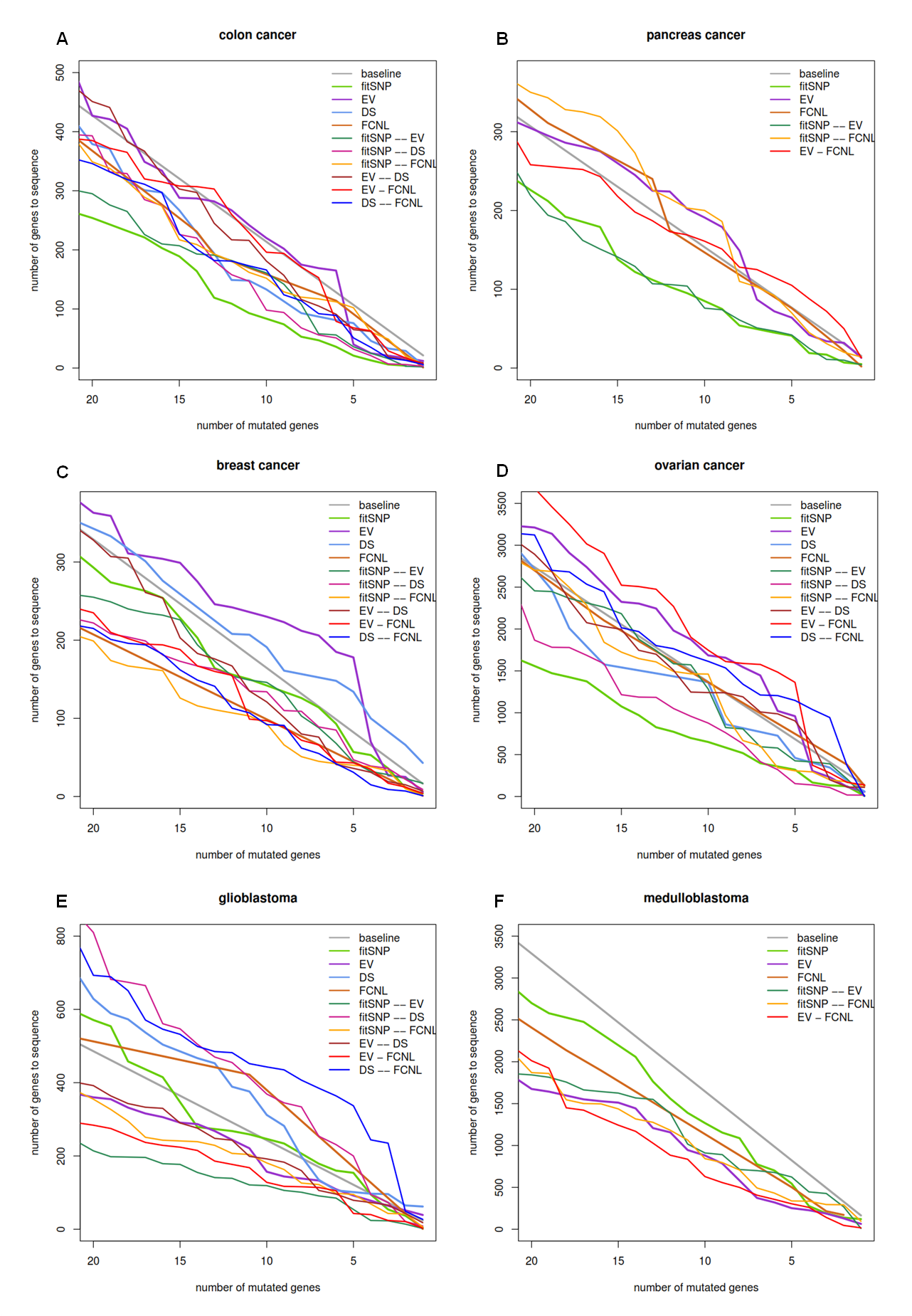

Supplement: Figure S2 — Visualization of the weighted ranking results for the top-100 ranked genes. The grey lines represent the ranking of the four different tumor types for the 10 prioritization strategies (4 single strategies and 6 combined strategies) and the baseline level. In red, the result of the brute force ranking algorithm is shown. The black line is where the baseline level is ranked across the different data sets. EV: expression variance; DS: dosage sensitivity; FCNL: frequency of copy number loss (TIF) [file pone.0031333.s002.tif]

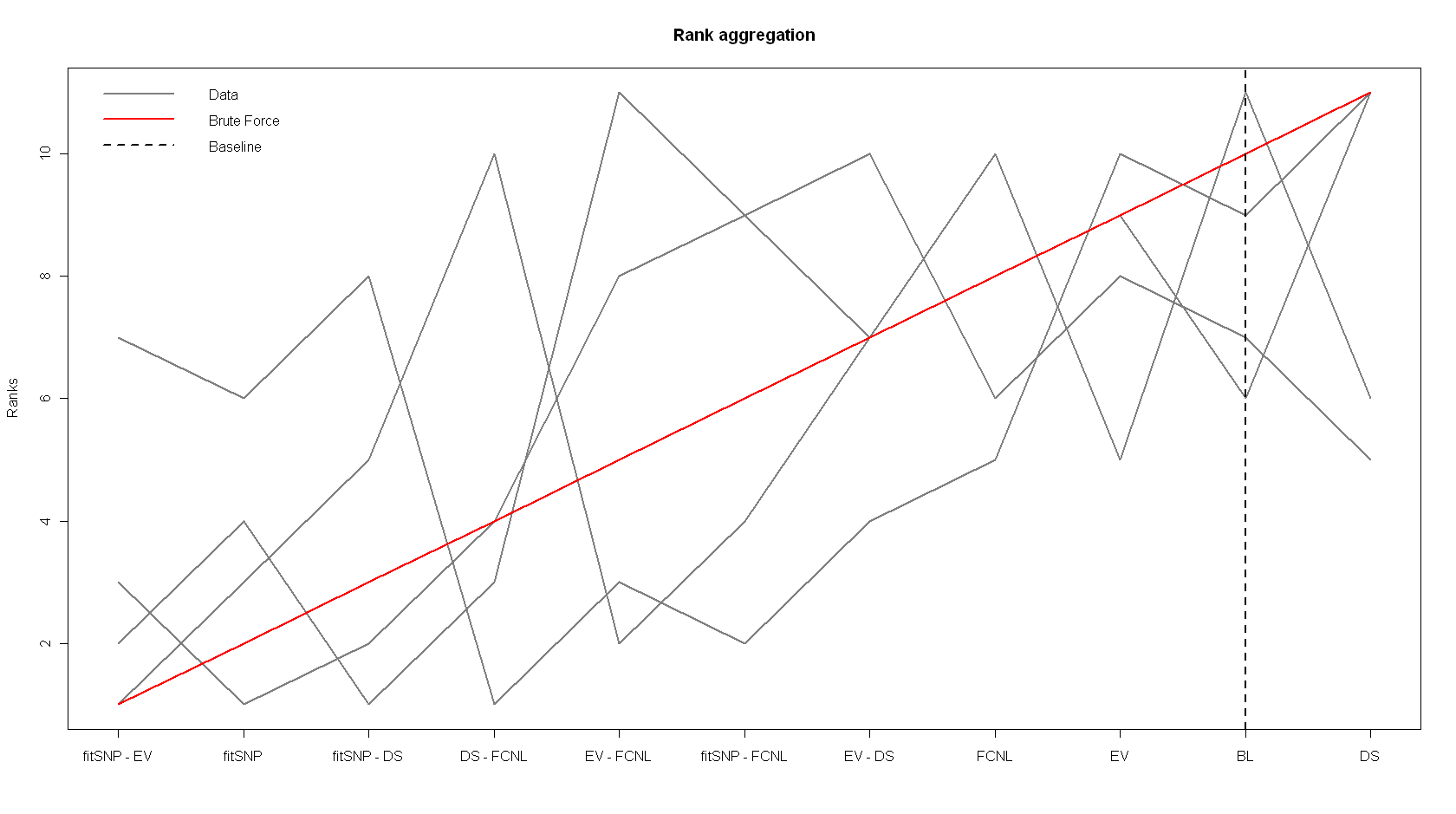

Supplement: Figure S3 — Plots of the number of mutated genes in relation to a certain number of top-ranked genes for the six cancer entities, including the combination strategies. (TIF) [file pone.0031333.s003.tif]

colon cancer

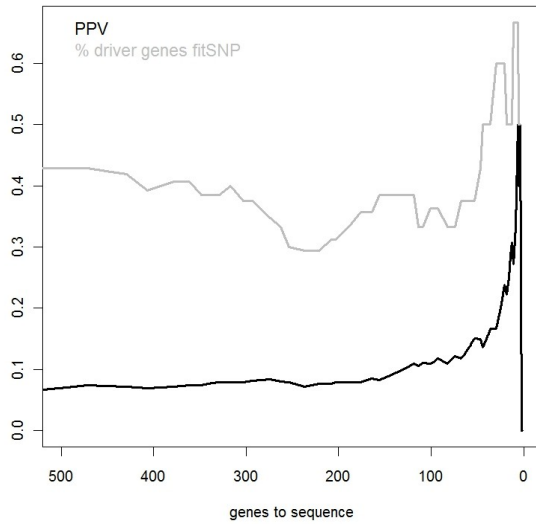

glioblastoma

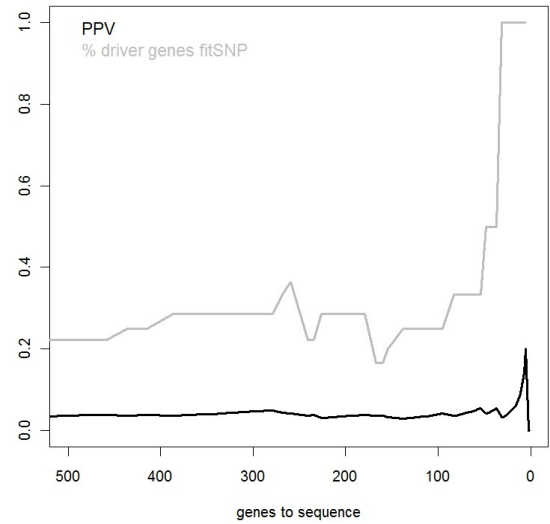

pancreas cancer

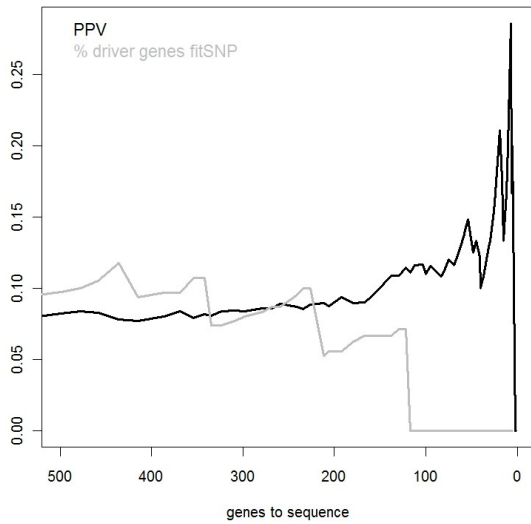

breast cancer

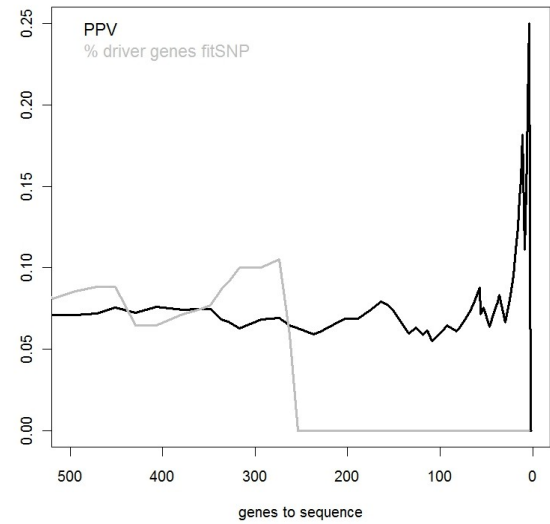

Supplement: Figure S4 — Plots of the percentage of mutated fitSNP genes that are found to be drivers. For colon cancer, glioblastoma, pancreas cancer and breast cancer, the PPV is plotted for the top 500 fitSNP genes (black line). The grey line represents the percentage of mutated fitSNP genes that are identified as driver genes according to the respective publications. Enrichment of identified driver genes can be seen in the top fitSNP genes in both colon cancer and glioblastoma, whereas in pancreas cancer and breast cancer this could not be confirmed. (PDF) [file pone.0031333.s004.pdf]
